# Adjunct Digital Interventions Improve Opioid-Based Pain Management: Impact of Virtual Reality and Mobile Applications on Patient-Centered Pharmacy Care

**DOI:** 10.3389/fdgth.2022.884047

**Published:** 2022-06-13

**Authors:** Hayam Y. Giravi, Zack Biskupiak, Linda S. Tyler, Grzegorz Bulaj

**Affiliations:** ^1^University of Utah College of Pharmacy, Salt Lake City, UT, United States; ^2^Department of Medicinal Chemistry, L.S. Skaggs College of Pharmacy, University of Utah, Salt Lake City, UT, United States; ^3^Department of Pharmacotherapy, L.S. Skaggs College of Pharmacy, University of Utah, Salt Lake City, UT, United States

**Keywords:** pharmacotherapy, analgesics, mHealth, smartphone apps, therapeutic video games, serious video games, opioid epidemic, health care

## Abstract

Digital therapeutics (DTx, mobile medical apps, software as a medical device) are rapidly emerging as clinically effective treatments for diverse chronic diseases. For example, the Food and Drug Administration (FDA) has recently authorized a prescription virtual reality (VR) app for treatment of moderate to severe low back pain. The FDA has also approved an adjunct digital therapy in conjunction with buprenorphine for opioid use disorder, further illustrating opportunities to integrate digital therapeutics with pharmacotherapies. There are ongoing needs to disseminate knowledge about advances in digital interventions among health care professionals, policymakers, and the public at large. This mini-review summarizes accumulating clinical evidence of digital interventions delivered *via* virtual reality and mobile apps to improve opioid-based analgesia. We identified relevant randomized controlled trials (RCTs) using Embase and PubMed databases which reported pain scores with a validated pain scale (e.g., visual analog scales, graphic rating scale, numeric rating scale) and use of a digital intervention in conjunction with opiates. Among identified RCTs, the majority of studies reported improved pain scores in the digital intervention group, as compared to “treatment as usual” group. Our work suggests that VR and mobile apps can be used as adjunct digital therapies for pain management. We discuss these findings in the context of how digital health technologies can transform patient-centered pharmacy care.

## Introduction

Pain management is a complex, multifaceted challenge that has become a major public health crisis, with an estimated 126.1 million US adults suffering from pain (1). In 2016, over 60 million patients filled or refilled one or more prescriptions for opioid analgesics (1). Although opioid-based analgesia is frequently used to treat both acute and chronic pain, health care professionals (physicians, physician assistants, pharmacists, and nurses) have limited knowledge on opioid analgesic therapies (2). In addition to inadequate pain relief, the use of opioids for pain management is challenged by significant adverse effects including physical dependence, tolerance, sedation, dizziness, constipation, nausea, vomiting, and respiratory depression (3). Trends in opioid prescription and the associated mortality continue to be problematic not only in the US, but also in other countries (4, 5). Multimodal approaches for pain management such as combination therapy with both nonpharmacological means in addition to traditional pharmacological therapeutics can be effective in achieving optimal control of pain (6, 7). Many aspects of pain management and the opioid epidemic may effectively be addressed by shifting clinical practice to using more non-pharmacological and non-invasive treatments (8), including “digital analgesics” interventions (9–13) and mobile apps to support opioid tapering (14, 15).

Digital health technologies encompass diverse software-based tools which can improve health and therapy outcomes for many chronic diseases. Digital therapeutics (DTx), also known as mobile medical applications, are software-based interventions intended to treat specific medical conditions (16–18). To provide evidence-based therapies, DTx receive marketing authorization (software as a medical device, or SaMD) from regulatory agencies. In the US, the FDA has approved and cleared several digital therapeutics for the treatment of diabetes (type 1 and 2), ADHD, asthma, COPD, chronic low back pain, chronic insomnia, substance use disorder and opioid use disorder. It is also noteworthy that two non-profit organizations, namely The Digital Medicine Society (www.dimesociety.org/) and The Digital Therapeutics Alliance (dtxalliance.org/) are dedicated to advance and promote this rapidly evolving branch of digital health.

Pioneering work on the SnowWorld virtual reality (VR) video game for burn patients illustrates early efforts to bring digital interventions for pain to clinical practice (19–22). There has been an increasing number of clinical studies on VR and mobile apps to improve pain management and relief (6, 9–11, 23–39). For example, a 12-week RCT of a multidisciplinary back pain mobile app (Kaia) showed significant reduction of pain intensity in patients with non-specific low back pain (40). In 2020, the FDA granted a breakthrough medical device designation to a VR app, RelieVRx (previously named EaseVRx), for treatment of intractable low back pain and treatment-resistant fibromyalgia. In 2021, RelieVRx received the FDA authorization for marketing a prescription virtual reality app for treatment of moderate to severe low back pain (41). These advances in digital interventions highlight opportunities for their use as adjunct therapies in combination with diverse analgesic drugs.

To the best of our knowledge, there are currently no review studies focused on effects of digital interventions on opioid-based pain management. Given the increasing number of clinical studies on VR and mobile apps for pain, there is a need for systematic reviews and meta-analyses (SR/MA) of the impact of DTx on different types of pain in combination with analgesic medications. The objectives of this mini-review are: (1) to summarize findings from currently published RCTs focused on adjunct digital interventions (VR and mobile apps) for opioid-based pain management, and (2) to encourage future SR/MA studies on adjunct digital interventions for pain in combination with specific analgesic drugs, including opioids, NSAIDs and others. We further discuss our findings in the context of how digital therapeutics can impact patient-centered pharmacy care.

## Adjunct Digital Interventions For Opioid-Based Analgesia

In order to identify adjunct digital interventions for pain management in conjunction with opioid-based analgesia, EMBASE and PubMed databases were searched for relevant RCTs, systematic reviews and meta-analyses. Database search with keywords “pain,” “acute pain,” “chronic pain,” “cancer pain,” “burn pain,” “postoperative pain,” “pain management,” “virtual reality,” “VR,” “web-based,” “phone app,” “mobile app,” “opioid” and “digital therapeutics” identified nine RCTs which met the following inclusion criteria: (1) reported digital interventions were compared to pharmacological interventions alone, (2) reported pain scores with a validated pain scale (e.g., visual analog scales, graphic rating scale, numeric rating scale), and (3) reported use of concomitant opioids. Studies that did not explicitly report use of opioids or use of a validated pain scale were excluded. In addition to searching the databases, we also examined RCTs evaluated in recent systematic reviews and meta-analyses on digital interventions for pain for those clinical studies that matched inclusion/exclusion criteria mentioned above (30, 32–34).

As summarized in Table 1, our search yielded nine RCTs which met inclusion and exclusion criteria. A majority of RCTs examined effects of digital interventions in burn pain patients (a total of *n* = 227), whereas two studies were focused on cancer pain. Regarding types of digital interventions, a vast majority of studies used VR apps. Eight studies demonstrated significant reduction in one or more pain outcomes (19, 42–45, 47–49), whereas one RCT reported no significant changes in pain intensity, as compared to the control groups. Based on the RCTs listed in Table 1, these findings suggest that adjunct digital interventions can improve pain scores or reduce medication use in opioid-based analgesia.

**Table 1:** Summary of randomized controlled trials of digital interventions in patients with acute or chronic pain.

**Author**	**Study design; Duration or number of sessions**	**Pain type;** **Pain scale**	**Population (*n*), intervention, and comparator description**	**Concomitant medication(s)**	**Results**
Bani Mohammed et al. (42)	Prospective RCT 40 sessions	Cancer Pain VAS^a^	**Population (*****n*** **=** **80):** Women (ages 30–70 years) with breast cancer **Intervention (*****n*** **=** **40):** VR (Ocean Rift interactive game or Happy Place non-interactive video) plus morphine **Comparator (*****n*** **=** **40):** Oral or intravenous morphine alone	IV or oral morphine	One session of adjunct VR resulted in a significant reduction in pain scores when compared to morphine alone (mean post-VAS score: iVR 0.33 vs. control 4.84; *p* <0.001)
Carrougher et al. (19)	Within-subject RCT 78 sessions	Burn Pain GRS^b^	**Population (*****n*** **=** **39):** Adult burn patients (ages 21–57 years) who required PT **Intervention:** VR (SnowWorld) with pharmacological analgesia during PT **Comparator:** Pharmacological analgesia alone	Oral methadone or OxyContin and a preprocedural short-acting opioid (e.g., oxycodone)	Adjunctive VR significantly reduced worst pain scores by 27% (VR 40 ± 30 vs. control 55 ± 25; *p* = 0.004)
Hoffman et al. (43)	Within-subject RCT 22 sessions	Burn Pain GRS^a^	**Population (*****n*** **=** **11):** Pediatric and adult patients (ages 9–40 years) with burns requiring hospitalization **Intervention:** VR (SnowWorld) with pharmacological analgesia during dressing changes **Comparator:** Pharmacological analgesia alone	Standard opioid analgesics and benzodiazepines	Mean pain ratings were lower with adjunct iVR when compared to the control group for all 3 pain measures (worst pain, time spent thinking about pain, and pain unpleasantness); differences were all statistically significant (*p* <0.05)
Hoffman et al. (44)	Within-subject RCT 24 sessions	Burn Pain VAS^b^	**Population (*****n*** **=** **12):** Adult burn patients (ages 19–47 years) **Intervention:** VR (SpiderWorld) with pharmacological analgesia during PT **Comparator:** Pharmacological analgesia alone	Long-acting opioids (typically OxyContin)	All 12 participants reported statistically significant less pain with adjunct VR distraction (worst pain: VR 19.92 vs. control 42, *p* = 0.002; average pain: VR 14.67 vs. control 36.33, *p* = 0.002)
Maani et al. (45)	Within-subject RCT 24 sessions	Burn Pain GRS^a^	**Population (*****n*** **=** **12):** US soldiers (ages 20–27 years) with burn wounds **Intervention:** VR (SnowWorld) with pharmacological analgesia during wound debridement **Comparator:** Pharmacological analgesia alone	Fast acting opioids and/or ketamine	Significant difference in mean worst pain scores >7 (iVR 5.67 vs. control 8.33; *p* = 0.043); no significant difference between groups for mild to moderate pain (VR 4.17 vs. control 3.33)
Morris et al. (46)	Within-subject RCT 22 sessions	Burn Pain NRS^a^	**Population (*****n*** **=** **11):** Adult burn patients (ages 23–54 years) undergoing physiotherapy **Intervention:** VR (eMagin Z800 3DVisor; game: Chicken Little) with pharmacological analgesia during PT **Comparator:** Pharmacological analgesia alone	Morphine and acetaminophen/ codeine (Dolorol Forte) to all eligible subjects; ibuprofen was given to two subjects	No significant difference in pain reduction between both groups (mean difference = 2.09; 95% CI-0.67 to 4.85, *p* = 0.13)
Schmitt et al. (47)	Within-subject RCT 1 to 5 days	Burn Pain GRS^b^	**Population (*****n*** **=** **54):** Hospitalized pediatric (ages 6–19 years) burn patients undergoing physical therapy **Intervention:** VR (SnowWorld) with pharmacological analgesia during PT **Comparator:** Pharmacological analgesia/sedation alone	Oral opioid (e.g., hydromorphone, fentanyl lozenge) +/− oral benzodiazepine (e.g., midazolam)	Significant reduction in cognitive (decreased by 44%), affective (decreased by 32%), and sensory pain (decreased by 27%) with adjunct immersive VR (*p* <0.05)
Sharar et al. (48)	Within-subject RCT 146 sessions	Burn Pain GRS^b^	**Population (*****n*** **=** **88):** Pediatric and adult patients (ages 6–65 years) who required postburn PT **Intervention:** VR (SnowWorld) with pharmacological analgesia during PT **Comparator:** Pharmacological analgesia alone	Systemic opioid and/or benzodiazepine	Significant decrease in worst pain intensity scores in the VR group (VR 43.5 ± 3.5 vs. control 54.2 ± 3.1; *p* = 0.003)
Yang et al. (49)	Prospective RCT 4 weeks	Cancer Pain NRS^a^	**Population (*****n*** **=** **58):** Adults (ages 18–75 years) with cancer-related pain **Intervention (*****n*** **=** **31):** Pain Guard mobile app **Comparator (*****n*** **=** **27):** Standard pharmaceutical care	Oxycodone, morphine, methadone, and/or tramadol	Pain Guard significantly decreased the frequency of breakthrough cancer pain (Pain Guard: median 3, IQR 2–7 vs. control: median 13, IQR 9.5–14, *p* <0.001) and lead to a higher rate of pain remission (*p* <0.001) with fewer adverse events reported

*Only RCTs which compared digital interventions with pharmacological interventions alone are included in this table*.

*GRS, graphic rating scale; IQR, interquartile range; iVR, immersive virtual reality; n, number of participants; NRS, numerical rating scale; PT, physical therapy; RCT, randomized controlled trial; VAS, visual analog scale; VR, virtual reality; vs., versus*.

*^a^Measured on a 0- to 10-cm scale*.

*^b^Measured on a 0- to 100-mm scale*.

Two additional RCTs investigated digital interventions in pain patients taking opioid analgesics, but they did not meet all three inclusion criteria (the comparator was not pharmacological treatment alone) (13, 50). In one RCT examining digital intervention in breast cancer surgery patients, the treatment group showed significant reduction of time (by **5** days) toward cessation of opioid medications, as compared to the control group (digital health education) (13). In another RCTs, VR app intervention (as compared to standard iPad use) did not change postoperative pain scores nor opioid consumption in pediatric patients (50). As discussed below, with more ongoing RCTs studying digital interventions and opioid-based analgesia in pain patients, our results justify near-future SR/MA study to evaluate clinical efficacy of adjunct VR and mobile apps in conjunction with analgesics to improve pain management.

## Discussion

There are ongoing needs to mitigate the opioid crisis in the United States (51, 52). To increase awareness about potential benefits of digital interventions for pain management, this mini-review project focused on whether virtual reality and mobile apps can improve opioid-based analgesia. Our findings suggest that VR applications can offer clinical-grade interventions for opioid-sparing pain management, and are in accord with conclusions from a recent systematic-review and meta-analysis that “*Virtual reality is an effective pain reduction measurement added to analgesics for burn patients undergoing dressing change or physical therapy*.” (32). The FDA authorization to market RelieVRx as a prescription virtual reality pain treatment further emphasizes opportunities to combine digital interventions with analgesics (41). It is noteworthy that clinical evidence for digital interventions in pain management is still limited and needs additional multi-center RCTs to validate their clinical efficacy and effectiveness in patients with various pain conditions (30–34, 53).

As shown in Table 2, there are several VR and mobile applications currently available for patients and health care providers as tools for improving pain management. RelieVRx has received the FDA authorization (through de novo regulatory pathway) to be marketed as a prescription virtual reality pain treatment for adult patients with chronic low back pain (41). Kaia Health is a mobile app intended for adults with acute or chronic, non-specific musculoskeletal pain, which received class II medical device status in Europe, while is marketed in the US under the FDA enforcement discretion. Flowly VR and biofeedback app is presented as “opioid-sparing pain management device” (www.flowly.world/), but to the best of our knowledge, Flowly has not received the FDA authorization as a medical device, as of writing this mini-review. While digital health technologies are rapidly evolving and expanding, we believe that this article will encourage health care professionals to explore opportunities to integrate digital interventions with pharmacotherapies for improved pain management.

**Table 2:** Examples of mobile and VR applications for pain management.

**Developing** **company**	**Available applications**	**Description of application**	**Mechanism of action**	**Clinical data**
AppliedVR	*RelieVRx*; *EaseVRx*	Marketed for the treatment of moderate to severe low back pain. Manage pain *via* immersive experience, guide patients to desirable clinical outcomes. Opioid sparing clinical treatment. Participants in their VR intervention for 2 weeks endorsed reduced pain catastrophizing scores as well as reduced overall pain.	Theories stemming from Cognitive Behavioral Therapy (CBT) employed in tandem with VR.	Garcia et al. (54) Garcia et al. (9) Spiegel et al. (55)
BreatheVR	*BreatheVR*	BreatheVR is a companion application for the Gear VR and Oculus GO VR setups. 8 of 10 participants in the initial pilot study all reported significant reductions in pain after only short periods of time using BreatheVR.	Deep breathing techniques in combination with a specifically designed relaxation VR landscape.	Mevlevioglu et al. (56)
Flowly	*Flowly*	The Flowly mobile application manages pain using theories from biofeedback in combination with VR to encourage pain management for patients and teach lasting techniques. Participants in their initial trials reported lower pain scores, lower pain catastrophizing scores, and reported needing lower dosages of their opioid medication to manage pain following the intervention.	Use of VR in combination with Flowly's mobile application to teach techniques of biofeedback, promoting pain management.	Flowly (57)
Kaia Health	*Kaia Health*	Musculoskeletal pain care with the use of custom physical therapy or rehabilitation exercise programs. Users report reduction in pain symptoms, reduction in stress symptoms, and further benefits. Accessible, clinical grade PT from the comfort of home.	Use of AI algorithms to guide physical therapy and rehabilitation sessions. Established PT methods such as progressive muscle relaxation. Used in combination with VR for best results.	Biebl et al. (58) Priebe et al. (25)

*CBT, cognitive behavioral therapy; VR, virtual reality; PT, physical therapy*.

Bringing digital interventions for pain to clinical practice is challenged by complexity of workflow in pain management (59). Mobile apps have been recognized as opportunities to improve pharmacy practice (60–63). Pharmacists often work in interdisciplinary care teams and make recommendations to both providers and patients about pharmacologic and non-pharmacologic interventions, including pain management (64–66). Given an important role of pharmacists in opioid stewardship and prevention of future opioid crisis (67, 68), we hypothesize here that pharmacists recommendations to integrate digital therapeutics with opioid-based analgesia will improve outcomes of opioid tapering programs (69–73). Recently, the Academy of Managed Care Pharmacy convened a forum that brought digital therapeutic innovators, payers, pharmacy benefit managers, and other key stakeholders to discuss the role of digital interventions as therapeutic options (74). While implementation of digital health technologies within health care systems is both inevitable and challenging (75–77), it will be important for payers to consider their health care coverage, especially as more evidence emerges with the potential opportunity of lowering overall health care costs and increasing clinical outcomes. An initial cost-effectiveness analysis of the reimbursement rate for digital therapeutics for low back pain suggests economic benefits for health care in Germany (78).

Integration of digital interventions with drug-based therapies is illustrated by the FDA approval of a prescription adjunct digital therapeutic, namely reSET-O^®^ PDT, in conjunction with buprenorphine for opioid use disorder (OUD). This adjunct digital intervention was shown to improve therapy and health care outcomes, including cost-effectiveness (79–84). From the perspective of long-term therapy outcomes for chronic diseases, patients could benefit from research and development of both adjunct digital therapeutics and drug+digital combination therapies (using drug-device combination product regulatory pathway, where drug is combined with a mobile app approved as SaMD) (18, 85–89). Although drug+digital combination therapies offer a full integration of pharmacotherapy and non-pharmacological intervention, to the best of our knowledge there are no currently known such drug-device combination products. Other future prospects for improved patient-centered pain management may include integration of drug-based analgesia with patient education delivered *via* digital health technologies (29, 90, 91), and integration of digital health technologies with self-care and therapeutic home environment (92).

A limitation of this mini-review is a lack of systematic review methodology and meta-analysis, thus precluding to draw evidence-based conclusions on effectiveness of digital interventions for opioid-based analgesia. Given that clinical studies on digital interventions for reduction of opioid use in pain management is a very active area of research (e.g., from ClinicalTrials.gov: NCT04139564, NCT04010266, NCT03851042, NCT04273919, NCT04416555 and others), it is prudent to wait for more published results from all relevant RCTs. Another limitation of this project is a focus on opioid-based treatments, rather than on opioids and non-steroidal anti-inflammatory drugs (NSAIDs). This is due to a limited number of clinical studies which report use of specific pain medications when evaluating VR or mobile apps in pain management. We hope that despite these limitations, this mini-review will raise awareness on how digital interventions can improve patient-centered pharmacy care for pain and for other medical conditions.

Given complex and unmet needs to address the opioid crisis (52, 93), this review supports several actionable recommendations to be considered. Educating health care professionals, patients and policymakers about the FDA-approved VR and mobile apps for pain should be led by both patient advocacy groups (e.g., The American Chronic Pain Association and the US Pain Foundation) and professional organizations (e.g., The American College of Physicians and The American Academy of Neurologists). Integrated healthcare systems and hospitals can create VR simulation centers for patient education about their diagnosis and treatment options including digital interventions (94, 95). Educating pharmacists, nurses and physician assistants about digital health technologies will accelerate clinical workflow redesign to incorporate their “internal champions” roles in decision making for pain management (64–66, 77, 96). For opioid prescription and tapering for chronic pain, revisions and updates to the CDC guidelines and payer pharmacy coverage should include the use of digital therapeutics for pain relief and management (97). Lastly, increasing social media campaigns (98, 99), and direct-to-consumer advertising of VR and mobile apps for pain will expand public awareness about digital therapeutics, and will also impact prescribing practices in the future (100, 101).

## Conclusion

Our mini-review suggests that both VR and mobile apps can be used as adjunct digital therapies in conjunction with opioid-based analgesics for pain management. Such interventions, which are applicable to hospital, hospital at home and stay-at-home care, can improve patient-centered pharmacy care and opioid tapering outcomes. Rapidly evolving digital health technologies create opportunities to integrate pharmacotherapies with non-pharmacological treatments for pain, while regulatory approval of commercially available digital interventions as DTx for pain management is critical for reimbursement and health care implementation.

## Author Contributions

HG and GB: conceptualization, literature search and review, and manuscript writing. ZB: literature search and review and manuscript writing. LT: literature review and manuscript writing. All authors contributed to the article and approved the submitted version.

## Funding

GB acknowledges a research support by the ALSAM Foundation Grant.

## Conflict of Interest

GB is a founder and owner of OMNI Self-care, LLC, a health promotion company creating digital content for disease self-management and is a co-inventor on two issued US patents 9,569,562 and 9,747,423 “Disease Therapy Game Technology” and patent-pending application “Multimodal Platform for Treating Epilepsy”. These patents are related to digital health technologies, and are owned by the University of Utah. The remaining authors declare that the research was conducted in the absence of any commercial or financial relationships that could be construed as a potential conflict of interest.

## Publisher's Note

All claims expressed in this article are solely those of the authors and do not necessarily represent those of their affiliated organizations, or those of the publisher, the editors and the reviewers. Any product that may be evaluated in this article, or claim that may be made by its manufacturer, is not guaranteed or endorsed by the publisher.
